# Extracting value from total-body PET/CT image data - the emerging role of artificial intelligence

**DOI:** 10.1186/s40644-024-00684-w

**Published:** 2024-04-11

**Authors:** Lalith Kumar Shiyam Sundar, Sebastian Gutschmayer, Marcel Maenle, Thomas Beyer

**Affiliations:** Quantitative Imaging and Medical Physics (QIMP) Team, Medical University of Vienna, Vienna, Austria

## Abstract

The evolution of Positron Emission Tomography (PET), culminating in the Total-Body PET (TB-PET) system, represents a paradigm shift in medical imaging. This paper explores the transformative role of Artificial Intelligence (AI) in enhancing clinical and research applications of TB-PET imaging. Clinically, TB-PET’s superior sensitivity facilitates rapid imaging, low-dose imaging protocols, improved diagnostic capabilities and higher patient comfort. In research, TB-PET shows promise in studying systemic interactions and enhancing our understanding of human physiology and pathophysiology. In parallel, AI’s integration into PET imaging workflows—spanning from image acquisition to data analysis—marks a significant development in nuclear medicine. This review delves into the current and potential roles of AI in augmenting TB-PET/CT’s functionality and utility. We explore how AI can streamline current PET imaging processes and pioneer new applications, thereby maximising the technology’s capabilities. The discussion also addresses necessary steps and considerations for effectively integrating AI into TB-PET/CT research and clinical practice. The paper highlights AI’s role in enhancing TB-PET’s efficiency and addresses the challenges posed by TB-PET’s increased complexity. In conclusion, this exploration emphasises the need for a collaborative approach in the field of medical imaging. We advocate for shared resources and open-source initiatives as crucial steps towards harnessing the full potential of the AI/TB-PET synergy. This collaborative effort is essential for revolutionising medical imaging, ultimately leading to significant advancements in patient care and medical research.

## Introduction

Positron Emission Tomography (PET) has evolved from its initial role as a specialised research tool into an indispensable element in clinical diagnostics, thereby significantly enhancing our understanding of physiological and molecular activities within the human body. The integration of PET with computed tomography (PET/CT) marked a major advancement, melding metabolic imaging with anatomical detail to improve the accuracy and comprehensiveness of diagnostic assessments [1]. This evolution has broadened PET’s applications across various clinical disciplines, notably oncology, neurology, and cardiology [2]. The introduction of the Total-body (TB) PET/CT or extended axial field-of-view PET/CT systems represents a further enhancement, offering increased volume sensitivity and a synchronous view of the bodily processes [3–5]. This advancement not only has streamlined scanning efficiency but also has extended the scope of PET applications into unchartered territories in both clinical practice and medical research [6–9].

The ‘value’ of TB-PET extends well beyond its technological advancements. Its true value is encapsulated in the flexibility of imaging protocols as well as in novel applications in both clinical settings and research domains. Clinically, TB-PET is notable for its enhanced sensitivity and efficiency, enabling rapid imaging and low-dose protocols, as well as facilitating delayed and same-day dual-tracer imaging [10]. These attributes have already markedly improved diagnostic capabilities and patient experiences. While still in its early stages in clinical research, TB-PET has shown promise in exploring systemic interactions across organ systems and in fostering a more holistic understanding of the human body [11, 12].

To date, artificial Intelligence (AI) has already established a significant presence in the realm of radiology, and its impact is increasingly evident in nuclear medicine as well [13]. Over the years, AI has successfully integrated into the entire imaging workflow of PET, including aspects such as image acquisition, image reconstruction, data corrections, and data mining. In the context of TB-PET, the application of AI in augmenting TB-PET’s value is still in its nascent stages but is showing steady growth. Given that TB-PET generates dense and rich datasets, AI is expected to play a central role in transforming this data into meaningful insights. Moving beyond its previous role as a supplementary technology, AI is emerging as a fundamental component in the future of TB-PET research.

This manuscript aims to explore both the current and potential roles of AI in enhancing the functionality and utility of TB-PET. Central to this inquiry is an examination of how AI can not only streamline existing TB-PET procedures but also pioneer previously unexplored applications, fully capitalising on the technology’s advanced capabilities. This paper will also discuss the critical steps and factors necessary for the effective integration of AI into TB-PET research.

## Current applications of total-body PET: enhancing efficiency with AI

The clinical community has shown ardent interest in TB-PET, largely because of its greater sensitivity compared to traditional short-axial field-of-view PET/CT systems. This enhanced volume sensitivity facilitates two key imaging options: rapid acquisitions with conventional dose injection and low-dose imaging over standard acquisition times. The first approach allows for swift, comprehensive imaging, essential for a detailed assessment of disease in a single bed position. Conversely, the latter option allows for the distribution of radiation dose over time, enabling longitudinal studies for more detailed disease observation and characterization. Furthermore, TB-PET’s heightened sensitivity also permits single-day, dual-tracer imaging [10]. This approach entails sequential scanning utilising disparate tracers, thereby substantially optimising patient throughput and scanning logistics. Additionally, the advent of dynamic imaging in a single bed position, supplemented by vendors integrating direct parametric reconstructions into TB-PET systems, provides the opportunity for more nuanced characterization of oncological cases in clinical routine [14, 15]. But despite the advancements brought forth by TB-PET, it also introduces new challenges in the domain of clinical imaging.

### Revealing more, demanding greater quantification

Initial investigations using the uEXPLORER (United Imaging) with healthy subjects have demonstrated remarkable detail in PET images from extended scan durations (up to 20 min), showcasing clear delineation of vessel walls, spinal cord, and brain structures [5]. Subsequent clinical studies employing either the uEXPLORER or Siemens Quadra TB-PET/CT system have further demonstrated improvements in both image quality and lesion quantification [16–20]. Notably, delayed imaging techniques have been observed to enhance the contrast between lesions and their background while simultaneously reducing image noise [19, 21]. Beyond oncology, the efficacy of ultra-low-dose TB-PET in imaging cardiovascular conditions and autoimmune inflammatory diseases underlines its broad clinical utility [22, 23].

**Fig. 1:**
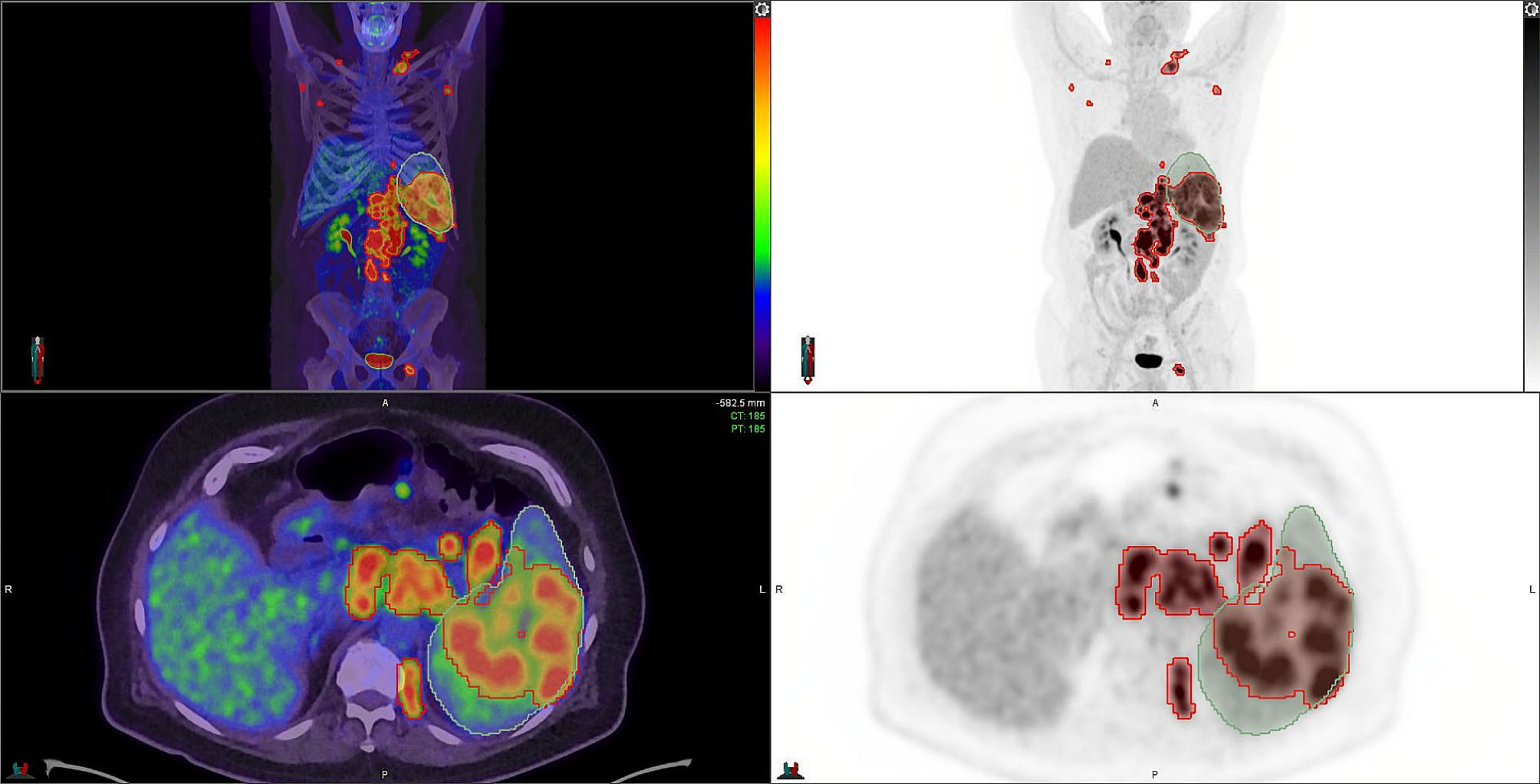
Multifaceted ^18^F-FDG PET Imaging Analysis of Follicular Lymphoma with AI-Assisted Tumor Detection. Presented here is a comprehensive visualization of follicular lymphoma characterized by diverse ^18^F-FDG uptake patterns across nodal and extranodal sites. The illustration captures the Molecular Imaging Tumor Volume (MTV) on ^18^F-FDG PET, delineated using the LION (Lesion Segmentation) algorithm, a native AI tool that identifies lymphoma lesions without the pre-setting of SUV thresholds. This intelligent segmentation excludes physiological uptakes in the kidneys, bladder, and brain for enhanced specificity in oncological imaging. Complementing this, the Multi-Organ Objective Segmentation (MOOSE) automatically defines organ contours, with a focus on the spleen in this instance. MOOSE enables the precise determination of the fraction of the spleen infiltrated by lymphoma, which is computed to be 56% of the total organ volume. The deployment of these AI algorithms for tumour and tissue segmentation provides a robust and reproducible quantitative assessment, offering novel prognostic insights into the extent and aggressiveness of follicular lymphoma

The comprehensive diagnostic capabilities offered by TB-PET, though invaluable, also come with risks of information overload for clinicians tasked with interpreting these complex scans. Traditionally, results have been derived through either visual assessment or labour-intensive manual segmentation, approaches that are increasingly inadequate given the breadth and volume of data provided by TB-PET. This is where AI-driven segmentation and detection tools become crucial, offering streamlined processing and interpretation of diverse biomarkers, from tumour loads (Fig. 1) and aortic wall uptake to systemic inflammations.

Currently, no single algorithm exists that can match the multi-label classification skills of a clinician across a variety of clinical scenarios. Nonetheless, significant progress has been made in tumour segmentation within ^18^F-FDG PET/CT imaging [24–26], driven by deep learning frameworks like nnU-Net [27] and MONAI Auto3DSeg [28], and supported by open-source datasets from initiatives such as AUTOPET [29] and HEKTOR [30]. Despite these advances, the challenge of algorithmic generalisation beyond specific training datasets persists. This limitation becomes particularly pronounced in total-body PET imaging, which encompasses a diverse range of clinical findings, from various tumour types to pathologies like inflammation and infection, particularly since these may coexist in individual patients. Consequently, developing individual algorithms for each distinct aspect within this domain is impractical, considering the vast diversity of data involved.

**Fig. 2:**
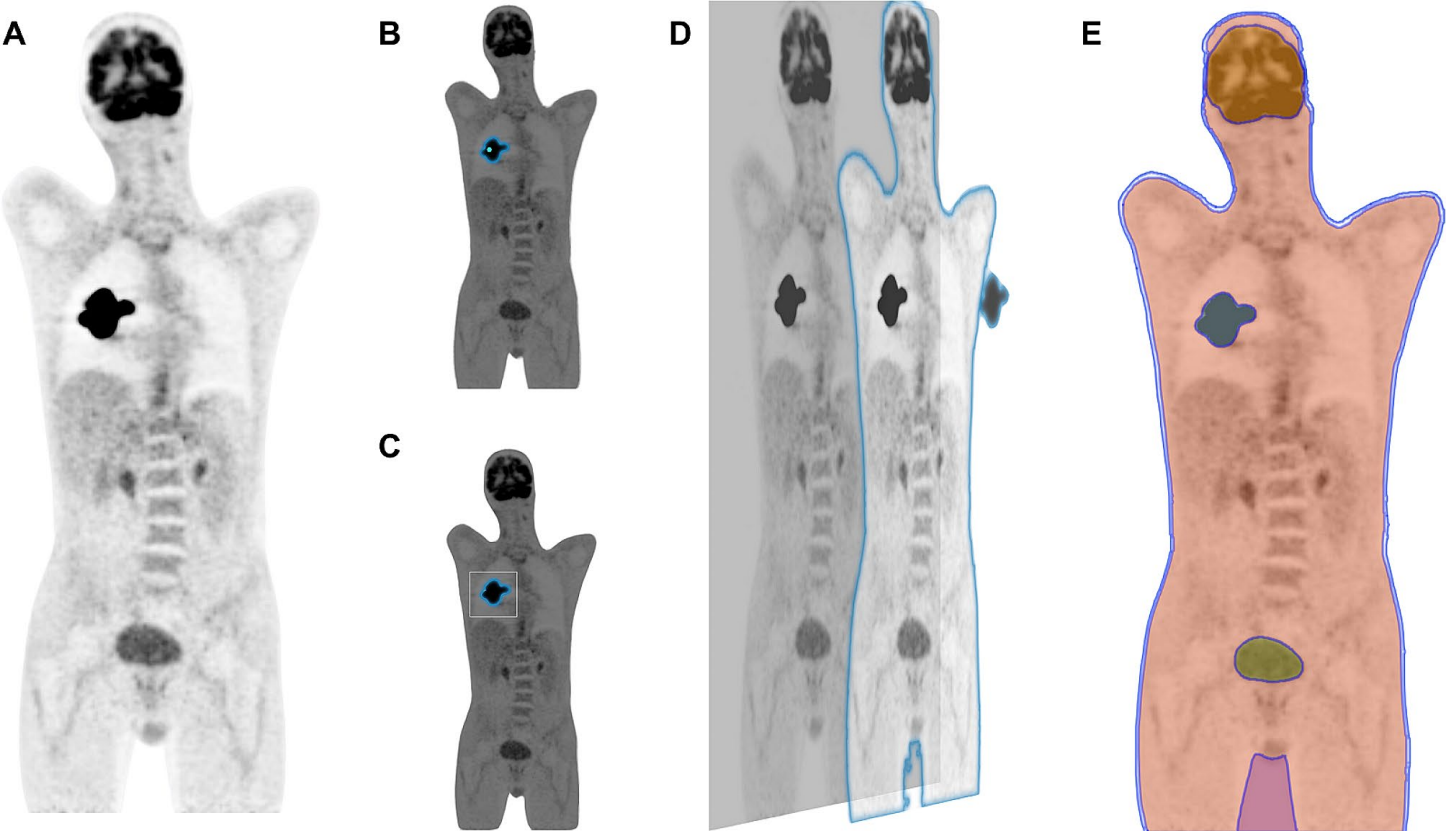
Comparative Visualization of ‘Segment Anything Model’ Performance on an Unseen 2D PET Image. Panel **A** displays the original PET image slice. Panel **B** illustrates the ‘Segment Anything Model’ executing point-based segmentation, pinpointing a singular region of interest (ROI). Panel **C** demonstrates the model applying a bounding box approach to encapsulate the ROI within a minimal rectangular boundary. Panel **D** presents the multi-mask segmentation capability of the model, initiated from a point-based prompt to discern multiple areas with varying intensities. Panel E showcases the fully autonomous segmentation proficiency of the ‘Segment Anything Model,’ delineating multiple ROIs without any manual prompts

In response to these challenges, the concept of foundational models offers a promising path forward. The success of vision models such as Meta’s ‘Segment Anything Model’ (SAM) [31] in general applications has inspired similar innovations in medical imaging. The Medical SAM (MedSAM), for instance, demonstrates the potential of these models to segment any specified area in medical imaging based on varied inputs like bounding boxes or points [32]. Interestingly, the native SAM is already capable of performing semantic segmentation on 2D PET images (Fig. 2), without any modification. A similar approach for 3D, tailored for PET imaging, could accelerate the analysis of complex TB-PET datasets. A foundational model that is agnostic to tracer or disease, would allow clinicians to efficiently segment and analyse diverse data, greatly facilitating the diagnostic process. The clinical impact of such a model could be profound, potentially automating the detection of key biomarkers such as Total Lesion Glycolysis (TLG) and Metabolic Tumour Volume (MTV), and efficiently quantifying systemic inflammation. This innovation holds the promise of becoming an essential tool in routine clinical practice, enabling more effective and efficient data mining from TB-PET imaging studies.

### In the radiation drama of total-body PET/CT: CT plays the lead

The substantial sensitivity increase in total-body PET/CT imaging has led to the advent of ultra-low-dose PET techniques, using as little as 1/20th of the standard dose while maintaining clinical image quality [33]. This advancement has broadened the scope for dose-optimised longitudinal imaging, with applications spanning various clinical areas [21, 34]. These include the development of new radiopharmaceuticals, monitoring of treatment responses, early detection of malignancy-related vascular complications, immune response imaging in infectious diseases, and paediatric imaging [21, 34–38]. The core concept involves dividing the total radiation dose across multiple scans to avoid additional radiation exposure. However, it’s important to note that in TB- PET/CT imaging, the primary source of radiation exposure is often not the PET component but rather the CT component. This aspect becomes particularly relevant in dual-time-point and dual-tracer studies, where patients undergo two CT scans [39, 40]. While CT is indispensable in providing essential anatomical details and enabling attenuation correction in clinical PET/CT studies, in the context of longitudinal imaging, either reducing the CT dose or omitting repeated CT scans could be beneficial. Such an approach aligns with the ‘As Low As Reasonably Achievable’ (ALARA) principle [41], supporting radiation safety initiatives like the ‘Image Gently’ campaign [42], which advocates for minimising radiation exposure, particularly in paediatric imaging. Researchers have already used AI in tackling this challenge, particularly in the context of attenuation correction. Sari et al. developed a deep learning-based method to create attenuation maps for PET scans without needing CT scans for correction [43]. Specifically, a convolutional neural network (CNN) was used to enhance initial µ-maps generated using a joint activity and attenuation reconstruction algorithm, showing promising results in enabling CT-free attenuation and scatter correction. This approach could be particularly useful in longitudinal imaging studies, where reducing or omitting CT scans can significantly lower patient radiation exposure while maintaining imaging quality. Likewise, Guo, Xue *et al* [44]. address a key challenge in CT-free PET imaging using deep learning (DL): the heterogeneity of tracers and scanners. They simplify this complex issue through domain decomposition, separating the learning process into low-frequency, anatomy-dependent attenuation correction and preserving high-frequency, anatomy-independent textures. This approach, trained with just one tracer on one scanner, showed robustness and effectiveness across various tracers and scanners, enhancing the potential for clinical translation of DL methods in PET imaging.

In another study by Hu et al., [45] an ultra-low-dose CT (ULDCT) reconstructed with an artificial intelligence iterative reconstruction algorithm (AIIR) was evaluated for use in ^18^F-FDG total-body PET/CT examinations. The study, including both phantom and clinical components, explored the feasibility of ULDCT (10 mAs) reconstructed with AIIR in comparison to standard-dose CT (SDCT) (120 mAs) using hybrid iterative reconstruction (HIR). The results indicated that while ULDCT-AIIR did not completely match the image quality of SDCT-HIR, it significantly reduced image noise and improved the signal-to-noise ratio (SNR), suggesting its potential application under specific circumstances in PET/CT examinations.

These advancements in AI for PET imaging not only enhance attenuation correction but also significantly increase the value of total-body PET by facilitating low-dose longitudinal imaging. This progression marks a pivotal step in maximising the clinical utility of PET imaging, offering more frequent and safer imaging options for patient monitoring and disease progression assessment, in line with minimising radiation exposure.

### Advancing disease characterization amidst growing data complexities

The utilisation of dual-tracer PET/CT imaging with ^18^F-FDG and ^68^Ga-PSMA has been instrumental in enhancing our understanding of tumour biology, specifically in terms of aggressiveness and differentiation. This approach, which combines ^18^F-FDG and ^68^Ga-PSMA tracers, has been implemented in preliminary studies using conventional PET/CT systems [39]. These studies have primarily focused on patient prognostic stratification. However, the integration of this dual tracer method into clinical routine has been limited. The primary challenges include increased radiation exposure and logistical complexities, such as organising scans on two separate days.

Recent advancements in TB-PET/CT have shown promising developments in addressing these challenges. Clinically viable protocols have been developed that allow for the sequential imaging of ^68^Ga-PSMA and ^18^F-FDG on the same day [40]. These protocols typically involve administering a standard dose of ^68^Ga-PSMA, followed by a low-dose ^18^F-FDG scan. Additionally, TB-PET/CT has been explored for dual-tracer PET/CT scans using ^18^F-FDG and FAPI tracers, offering insights into the tumour-associated microenvironment [10].

However, it is important to distinguish these practices from multiplexed PET imaging. Multiplexed PET imaging involves administering a mixture of tracers to the patient and employing advanced reconstruction techniques to isolate individual signals. This method offers two significant advantages: firstly, it facilitates a single imaging session without the need for a second CT or subsequent scan. Secondly, it enables voxelwise alignment, providing true spatial multiplexing. This capability is crucial for understanding the spatial heterogeneity of tumours, as the multiplexed image can simultaneously highlight various attributes of the tumour under investigation.

The advent of reconstruction-based multiplexing in PET/CT imaging represents a significant advancement in the field, offering a sophisticated approach to capturing complex biological processes in a single imaging session [46]. While this technique holds great promise, its implementation in clinical practice is not yet widespread, primarily due to its implementation complexity. However, an equally effective alternative can be achieved through the precise spatial alignment of dual tracer PET/CT images, a method that can be readily applied in current clinical settings with the aid of artificial intelligence (AI).

**Fig. 3:**
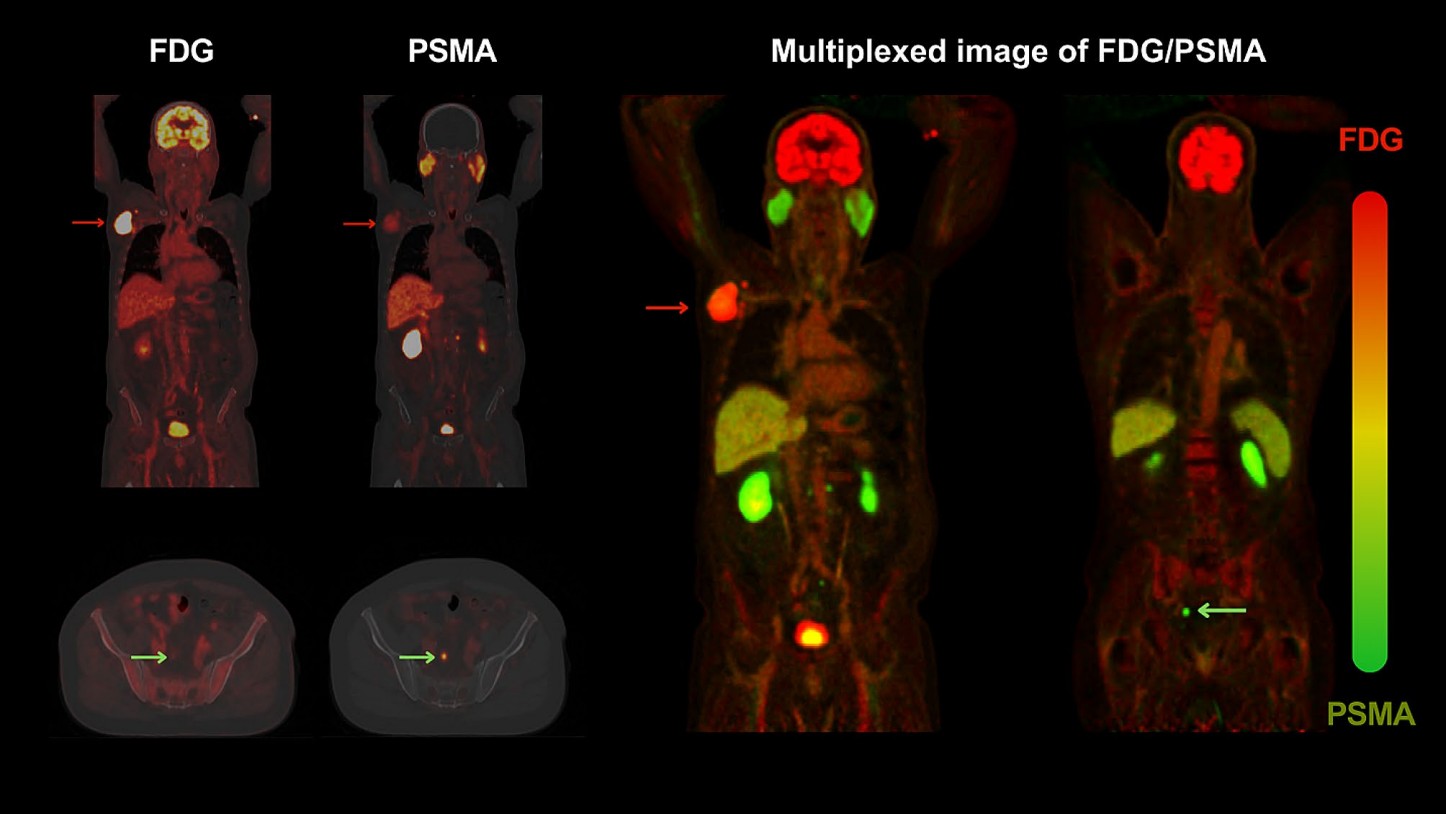
Characterising Oncological Heterogeneity through Multiplexed PET Imaging. The figure demonstrates the potential of multiplexed PET imaging technique on a patient with coexistent malignancies: ^68^Ga-PSMA-positive/ ^18^F-FDG- negative prostate cancer (refer to PET/CT axial slice) highlighted in green and ^18^F-FDG-avid metastatic melanoma (refer to coronal slice with prominent ^18^F-FDG uptake and mild ^68^Ga-PSMA uptake), highlighted with red arrows. Separate scans using ^18^F-FDG and ^68^Ga-PSMA tracers reveal distinct metabolic and molecular patterns corresponding to melanoma and prostate cancer, respectively. The composite image results from diffeomorphic algorithmic synthesis, assigning discrete chromatic channels—red for ^18^F-FDG and green for ^68^Ga-PSMA—to each radiotracer, thereby creating a composite image. This multiplexed image merges the two separate datasets into a single, integrated visual field. Manifestations of sole ^18^F-FDG uptake are visualized in red, ^68^Ga-PSMA uptake in green, and concomitant tracer accumulation is rendered in shades of yellow, indicating co-expression. ^18^F-FDG dominant malignancies in the multiplexed images are highlighted with red arrows, while ^68^Ga-PSMA dominant malignancies are highlighted with green arrows. This technique of image multiplexing, akin to multiplex histopathology, allows for a nuanced characterization of tumoural heterogeneity, providing an intuitive and single-image synopsis of the distinct pathophysiological processes at play

The spatial alignment of two distinct tracers in PET/CT imaging presents a notable challenge, as these tracers often exhibit varying activity distributions. A promising solution to this problem is aligning the corresponding CT images first and then transferring the derived motion fields to their PET counterparts. This technique, especially relevant in sequential dual-tracer scans performed on the same day, could effectively mimic the outcomes of reconstruction-based multiplexing, thus offering a ‘pseudo-multiplexing’ effect (Fig. 3).

Nevertheless, aligning the CT images is a complex task. Conventional diffeomorphic algorithms, despite their capability to handle large deformations, may not provide the necessary precision. Research has shown that augmenting these algorithms with dense segmentation maps can greatly enhance the accuracy of the motion fields, leading to more accurate alignment [47]. In this context, the use of advanced open-source CT organ segmentation tools such as MOOSE [48] and TotalSegmentator [49], which are based on the robust nnU-Net [27] AI framework, becomes crucial. These tools facilitate detailed whole-body segmentations, which, when integrated into the registration process, significantly improve alignment accuracy.

For the registration process, one can choose between classical diffeomorphic algorithms [50, 51], known for their effectiveness in computational neuroanatomy, or adopt contemporary learning-based algorithms like VoxelMorph [47]. Learning-based algorithms (e.g., VoxelMorph) offer a substantial advantage in terms of computational speed, as they eliminate the need for optimization during the inference process, unlike classical diffeomorphic algorithms, which are more computationally demanding. By leveraging AI, particularly in the alignment of dual-tracer TB-PET/CT images, we can approach the intricacies of tumour heterogeneity with a level of precision and detail akin to that achieved in multiplexing techniques used in immunohistochemistry.

**Fig. 4:**
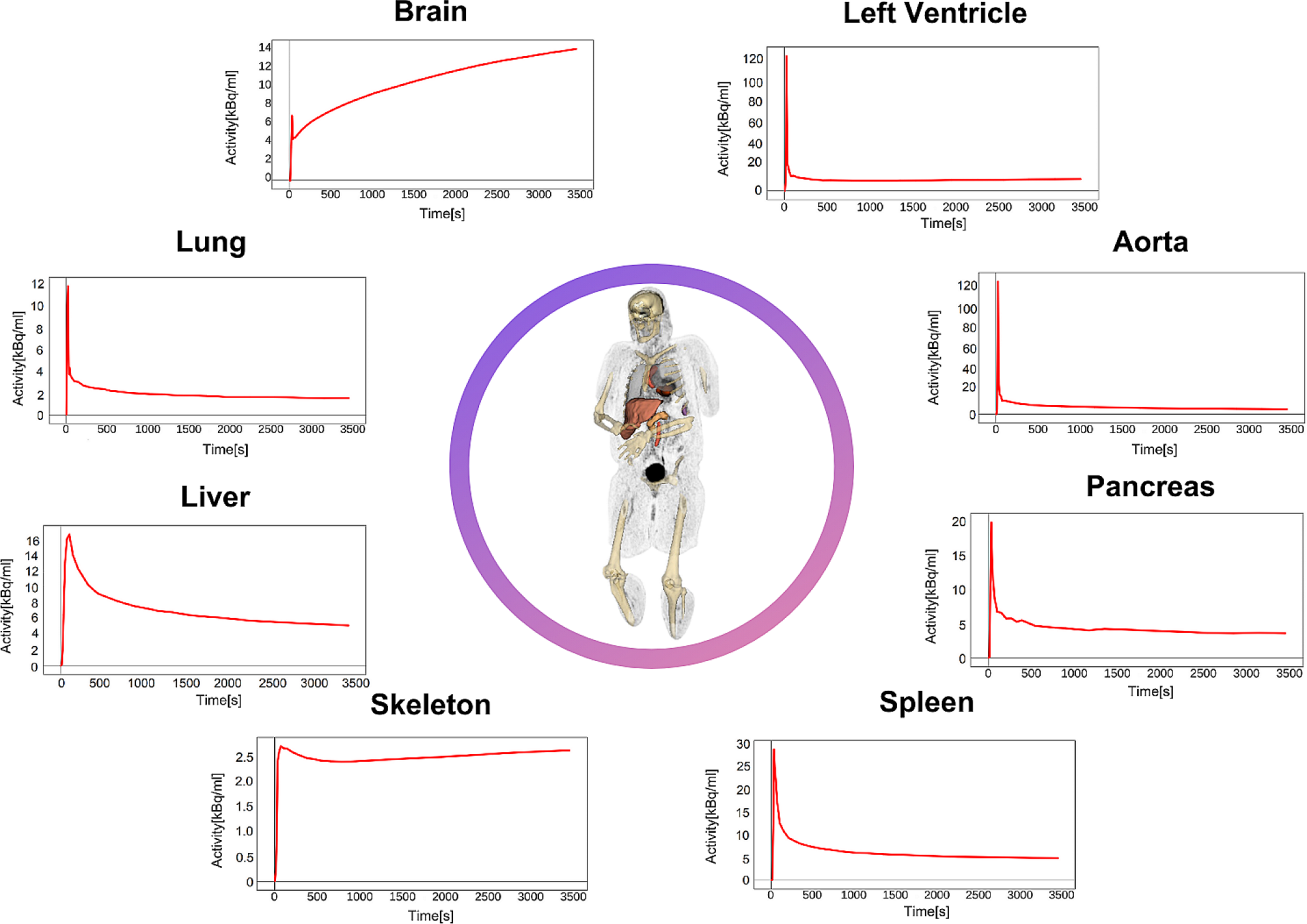
AI-Driven Multi-Organ Segmentation (MOOSE) for kinetic analysis in dynamic PET. This figure demonstrates an AI-assisted segmentation approach applied to dynamic PET/CT imaging for the extraction of time-activity curves (TACs) across multiple organs. Central is a PET image overlaid with segmented organs; surrounding it are graphs depicting TACs for the brain, left ventricle, aorta, lung, liver, pancreas, spleen, and skeleton. These curves are derived from dynamic PET scans post-segmentation and are instrumental in streamlining kinetic modeling and facilitating absolute quantification of tracer uptake, thus enhancing the precision of metabolic studies

Another emerging area of interest in TB-PET imaging is dynamic imaging, adding a temporal domain to the rich 3-D information intrinsic to PET. Recent research indicates that by analysing the raw time activity curves (TACs) of tumour regions, it is possible to assess the spatial heterogeneity within tumours [20, 52]. At the same time, kinetic modelling has garnered considerable attention as well. Research groups are exploring its applicability in oncology, particularly in ways to abbreviate the scan duration required for kinetic analysis [53, 54]. The objective is to extract kinetic parameters that provide a more nuanced understanding of the tumour under investigation. Nonetheless, these dynamic PET imaging techniques present several challenges. Characterising TACs requires precise tumour segmentation, and kinetic modelling depends on segmenting specific regions to determine the input function derived from imaging data (IDIF). Both tasks are labour-intensive and demand high precision. In this context, whole-body AI-based organ segmentation tools like MOOSE [48] and TotalSegmentator [49] prove invaluable. They facilitate the segmentation process for IDIF as both cover major input function regions, thereby streamlining kinetic modelling (Fig. 4). Employing a foundational AI model for tumour segmentation, as previously discussed, can significantly ease the extraction and analysis of tumour TACs. Integrating AI into TB-PET imaging workflows is essential to fully leverage dynamic imaging’s potential. Automating these processes reduces the manual and cognitive burden on clinicians, allowing them to concentrate more on interpretation and clinical decision-making.

## Novel applications from total-body PET: AI - a key enabler in generating value

### Comprehensive health assessment with total-body PET: a unified diagnostic approach

The capability of TB-PET/CT to simultaneously image the entire human body, combined with its high spatial and temporal resolution, presents certain unique opportunities. Recent research has demonstrated the potential of conducting sub-second image reconstructions with TB-PET/CT, closely mirroring the temporal resolution achieved by functional Magnetic Resonance Imaging (fMRI) [55]. These advanced capabilities in TB-PET/CT could herald a paradigm shift from traditional imaging (often colloquially referred to as “lumpology [56]”) to a renewed focus on PET’s fundamental strengths in assessing physiological and pathophysiological functions and processes. This capacity for high-temporal dynamic imaging across all organs promises to deliver a wealth of clinically relevant data, surpassing mere identification of pathologies and encompassing a comprehensive understanding of bodily functions. Measurements including first-pass cardiac ejection fractions, as well as pulmonary and renal perfusion assessments, may be derived through the analysis of finely sampled PET frames followed by voxel-level data evaluation, thereby providing an extensive assessment of health [37, 57]. In such studies, the definition of volume and motion correction is going to be crucial post-processing steps, essential for the generation of data that is both quantitative and useful. Furthermore, as previously discussed, the availability of various AI-based organ segmentation algorithms could prove to be indispensable in the facilitation of such research endeavours. Addressing motion correction in total-body PET presents a complex challenge, given the multifaceted nature of motion encountered in such settings. This includes gross body motion, respiratory and cardiac movements, as well as abdominal motion, with the motion profile varying from rigid structures like the brain to more deformable ones like the gut and bladder. Developing a motion compensation tool that effectively manages this range of motion profiles across various tracers poses significant difficulty.

Recent research has explored the application of diffeomorphic registration for total-body motion correction. For instance, Sun et al. [58] utilised Symmetric Normalisation [50] for whole-body motion correction in ^18^F-FDG PET/CT scans. In a similar vein, we introduced FALCON [59], a diffeomorphic algorithm optimised for speed and applied across various tracers to correct for total-**body** motion, albeit compromising the symmetric property of the algorithm for enhanced computational efficiency [51]. Notably, both these algorithms demonstrate limitations in correcting early frames (less than 2 min post-injection), where tracer dynamics undergo rapid changes critical for clinical perfusion parameters. The primary challenge here lies in the disparity of image content in these early frames, attributable to the swiftly changing tracer kinetics, which complicates the task of any correction algorithm.

To address this specific issue, the use of conditional Generative Adversarial Networks (GANs) has been proposed and effectively implemented in both brain [60] and total-body studies [61]. The objective of these networks is to create synthetic images resembling those of later frames from the early imaging data. However, a hurdle in this approach is the limited generalizability across different tracers, necessitating specific training for each type of tracer used.

With the emergence of generative AI models, such as diffusion models [62], there is potential to develop a more universal model capable of generalising across multiple tracers. Such a model could theoretically create a pseudo-late-frame image from early-frame data or transform all images into an intermediate synthetic form to facilitate motion correction, potentially overcoming the current limitations in early frame motion correction.

### Total-body PET with AI: a window into understanding normal physiology and health

Originally, PET imaging predominantly served as a tool for exploring physiological processes prior to its evolution into a clinical diagnostic instrument [63]. Concerns regarding radiation exposure have steered the medical community towards alternative modalities, notably MRI. However, the advent of TB-PET, coupled with advancements in minimising CT radiation exposure, has paved the way for ultra-low dose imaging. This innovation holds the promise of safely extending PET imaging applications to healthy populations, thereby broadening its utility in understanding normal physiology and non-malignant disease processes.

Comprehending normal physiology is paramount for the accurate interpretation of disease-related anomalies. Within the field of oncology, PET imaging has predominantly concentrated on tumours and their immediate surroundings. Nevertheless, the wider scientific consensus views cancer as a systemic condition, thus underscoring the need to extend focus beyond just the tumour’s locale. Observing the macroenvironment, particularly organ systems not directly compromised by tumour invasion, is crucial for a holistic understanding of cancer’s and therapies systemic and toxic effects [64, 65]. This approach is not only pertinent in oncology but may also hold significant relevance in elucidating musculoskeletal disorders and metabolic diseases, where systemic factors play a key role [66, 67].

The creation of a ‘normative database’ derived from healthy individuals is instrumental in facilitating the rapid systemic analysis of pathological cases. The notion of a normative database is well-established in medicine, providing clinicians with a benchmark of ‘normalcy’ for various parameters. This concept has been extensively applied in the realm of neuroimaging, where it has become a cornerstone in the identification of pathological conditions [68–71]. Extending this approach to total-body PET would allow for a similar utility in detecting systemic anomalies, offering a comprehensive reference point for distinguishing between normal and abnormal physiological states across the entire body.

Initial research in the realm of whole-body MRI, particularly under the scope of Imiomics [72], has laid the groundwork for establishing a proof-of-concept normative database. This database focused on quantifying average distributions of adipose and lean tissue within an asymptomatic population. Participants for this study were randomly selected from the general population, which meant that not all individuals were in perfect health. In this sample, 2% had diagnosed diabetes, 8% were known to have hypertension, and 4% were undergoing statin therapy. However, none of the participants suffered from severe diseases, such as cancer, myocardial infarction, stroke, heart failure, or chronic obstructive lung disease. Though not representative of a completely healthy cohort, this initial effort has laid the groundwork for developing a comprehensive total-body normative database, a crucial step in expanding the potential of PET imaging in systemic health assessment.

Aligning total-body PET images across individuals presents a significant challenge, particularly when compared to MRI. This difficulty arises from PET’s relatively lower resolution and variable tracer uptake characteristics. Nevertheless, it is feasible to utilise the accompanying CT images to facilitate alignment, subsequently transferring the deformable fields to their PET counterparts. In the process of constructing a normative database, the deformable alignment of healthy control images is a key step in creating a standard atlas of healthy individuals.

**Fig. 5:**
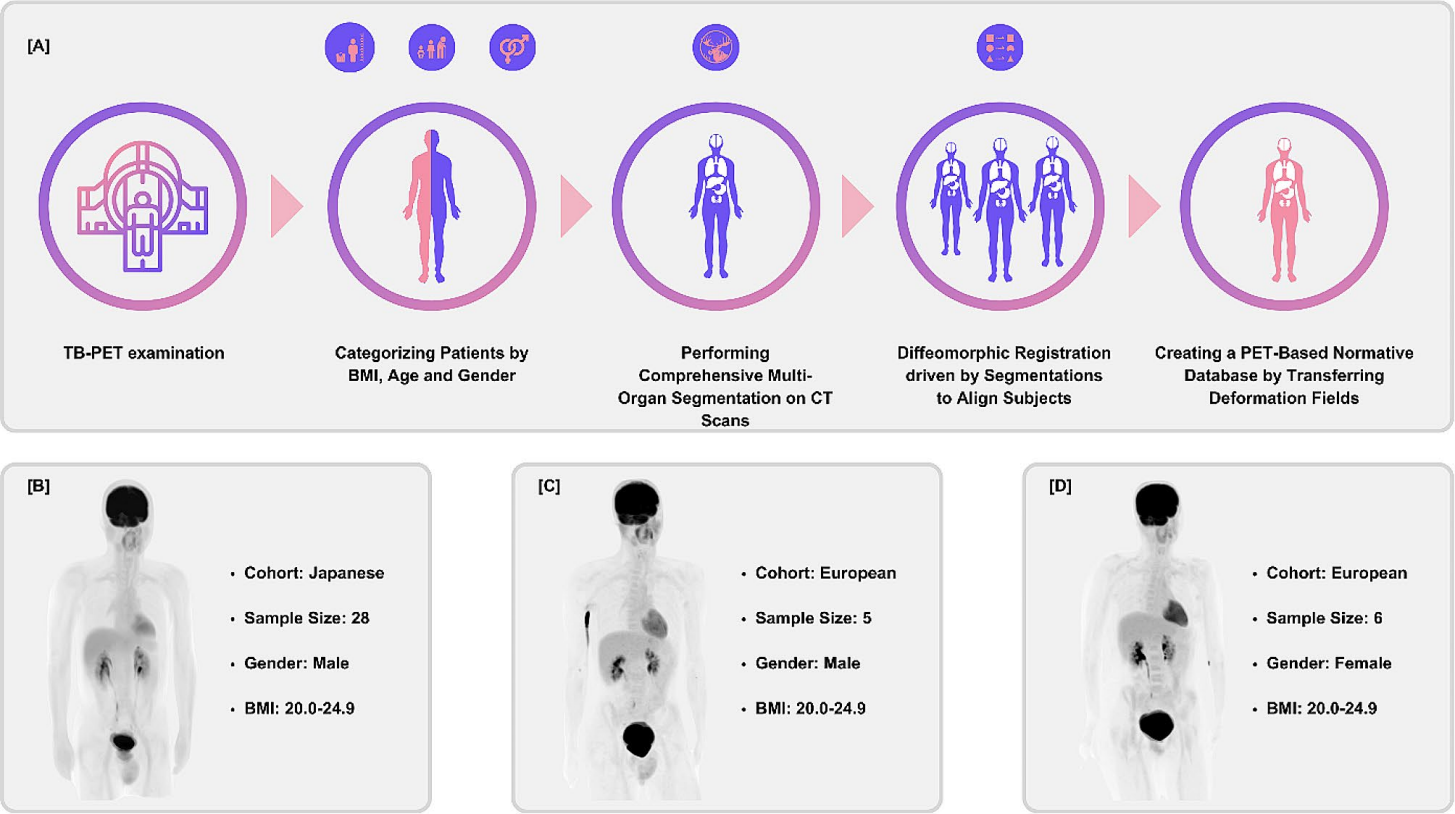
Methodology for Normative Database Construction from PET/CT Data. Panel [**A**] depicts the sequential process for establishing a normative database derived from PET/CT data. The protocol initiates with a TB-PET examination, followed by patient stratification according to BMI, age, and gender. The subsequent phase involves deriving detailed organ segmentations from CT scans. These segmentations then guide diffeomorphic registrations to align subjects across diverse cohorts. The derived deformation fields from the CT alignments are applied to the corresponding PET data, culminating in a comprehensive normative database. Panels [**B**], [**C**], and [**D**] display representative maximum intensity projections PET images from the database, segmented by cohort characteristics. Panel [**B**] exemplifies a Japanese male cohort with a BMI range of 20.0-24.9, while Panels [**C**] and [**D**] represent European cohorts, male and female, respectively, both within the same BMI range. The noticeable radiotracer uptake observed in the right arm of the normative template image in panel [**C**] is an artifact attributable to the initial administration site of the radiopharmaceutical. Such localized hyperactivity represents a procedural remnant rather than pathological significance. Each panel provides the cohort’s demographic and sample size data, reflecting the database’s population diversity

During this alignment process, two elements are of paramount importance: firstly, the alignment between subjects, and secondly, the segmentation that supports and enhances this alignment (Fig. 5). Recent advancements in AI, as discussed in the context of multiplexing, can greatly expedite this process. Tools producing dense segmentation maps, along with learning-based diffeomorphic methods like VoxelMorph [47], have the potential to significantly streamline the creation of normative databases. However, it is crucial to consider various confounding factors, such as age, body mass index (BMI), and gender, when developing these databases. Careful accounting for these variables is essential to ensure that the normative database accurately reflects the diversity and range of the healthy population [73]. This careful consideration is vital for the database to be a reliable and representative tool in clinical and research settings.

**Fig. 6:**
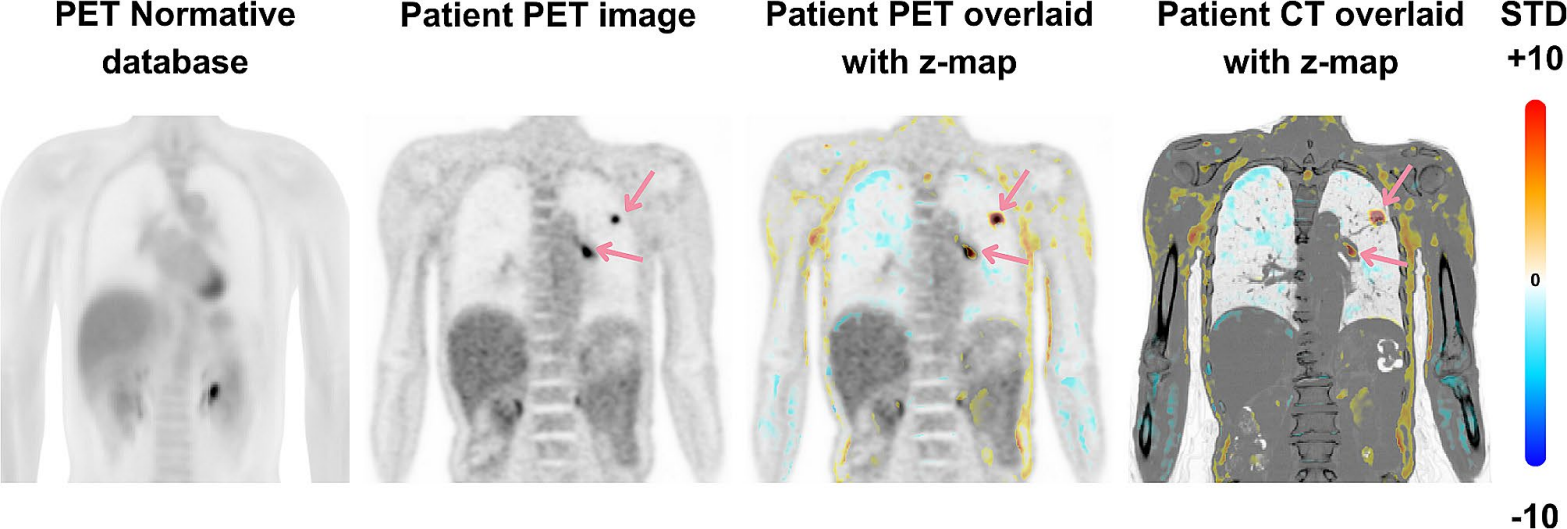
Metabolic aberration Analysis Using PET Normative Database. This figure presents a method for evaluating patient PET scans against a PET normative database. The first panel shows the averaged data from a healthy cohort forming the normative PET database, therefore providing a reference for typical tracer distribution. The second panel displays a lung cancer patient’s PET image, where abnormalities are indicated with arrows. The patient’s PET image is diffeomorphically aligned with the Normative database, and the deviations from the normalcy are calculated as z-maps. In the third panel, the patient’s PET data is overlaid with a z-map, highlighting deviations from the normative model. The fourth panel further overlays the z-map onto the patient’s CT image, offering anatomical context to the functional PET data. The colour scale on the far right indicates standard deviations from the normative mean, with warmer colours denoting higher deviations

The creation of a normative database via TB-PET not only paves the way for high-throughput screening in at-risk populations like lung cancer (Fig. 6) or breast cancer but also presents the opportunity to explore comprehensive assessments of physiological health and ageing effects throughout the body. Notably, achieving a crucial milestone in this endeavour is the reduction of the effective radiation dose to patients to levels below 1 mSv per scan. While ^18^FDG remains the clinical tracer-of-choice for many clinical applications, generating similar normative databases for additional tracers that are now routinely used in clinical practice, including PSMA and somatostatin receptor ligands, and emerging tracers, such as FAPI agents, will also be beneficial.

### Making sense of systemic information provided by total-body PET: AI

In previous sections, we have established that TB-PET/CT generates a comprehensive array of multi-dimensional systemic data. The extraction of meaningful insights from such data necessitates the adoption of robust analytical techniques, among which AI stands out as particularly suited for this task. Recent research initiatives have focused on delving into this multidimensional data to understand systemic effects across both healthy and pathological cohorts. These studies primarily utilise classical correlation analysis methods, which involve extracting organ-specific Standardised Uptake Values (SUVs) and generating correlation heatmaps within the cohorts under study. The fundamental aim is to identify variations in the resulting correlation maps [8, 11, 74, 75].

A notable advancement in this field was introduced by Sun et al. [11]., who proposed a novel methodology centred on the identification of individual deviations from normative patterns. This is achieved through a perturbation-based approach, where the baseline healthy correlation network is disrupted by integrating pathological cases, thereby facilitating the detection of individual anomalies. However, it is crucial to recognize that these studies typically involve relatively small sample sizes. Moreover, it is imperative to understand that these are correlation-focused studies that do not inherently imply causality.

In the context of analysing comprehensive datasets derived from TB-PET/CT scans, a multitude of methodological approaches are available to researchers. Key among these is the utilisation of robust computational frameworks such as scikit-learn [76], which facilitate the compilation of an extensive array of parameters from total-body datasets. These parameters include SUVs, kinetic parameters, and additional clinical data, such as volumetric measurements obtained from CT scans. Subsequent to parameter extraction, various machine learning algorithms can be employed to effectively differentiate between distinct groups, thus framing this analysis as a classification problem.

Alongside these conventional methodologies, the emergence of Automated Machine Learning (AutoML) represents a significant advancement in the field of medical image analysis. AutoML particularly enhances the automatic analysis of tabulated data from TB-PET scans. By automating critical tasks like model selection, hyperparameter tuning, and validation, AutoML renders advanced analytical techniques more accessible and efficient. Prominent frameworks in this domain include Google’s AutoML [77], H2O AutoML [78], and TPOT (Tree-based Pipeline Optimization Tool) [79]. Google’s AutoML is notable for its user-friendly interface and powerful algorithms that adeptly handle complex data structures, making them suitable for researchers with varying levels of programming expertise. H2O AutoML is acclaimed for its efficiency in rapidly producing high-quality models. Conversely, TPOT leverages a genetic programming approach to optimise machine learning pipelines, ensuring optimal model adaptation for specific datasets.

The incorporation of these AutoML frameworks into the analysis of total-body PET data substantially streamlines the identification of relevant features and patterns. By automating the more labour-intensive aspects of model building, researchers can devote greater attention to interpreting results and extracting clinically relevant insights. Additionally, the iterative model refinement and adaptability to new data inherent in AutoML, ensure that analyses remain at the forefront of medical dataset evolution.

To further enhance the transparency and interpretability of these algorithms, the application of explainable AI methods is advantageous. Techniques such as SHAP [80] (SHapley Additive exPlanations) and LIME (Local Interpretable Model-agnostic Explanations) [81] elucidate how individual features contribute to specific algorithmic decisions. This clarity is instrumental in elevating the interpretability of the results.

However, when employing these machine learning techniques, it is crucial to exercise caution to circumvent issues like overfitting and underfitting. A commonly overlooked yet critical aspect is the sample-to-feature ratio [82, 83]. Maintaining a minimum ratio of 10:1 is widely recommended, serving as a reasonable benchmark to ensure the robustness and reliability of the model’s performance.

The recent advancements in deep learning open promising avenues for mining TB-PET datasets, especially through the creation of embeddings [84]. Utilising deep learning architectures like convolutional neural networks (CNNs) [85] or Vision Transformers [86], three-dimensional PET images can be transformed into high-dimensional vector embeddings. These embeddings have the potential to concisely capture the comprehensive physiological and metabolic profiles of patients, offering a distilled yet information-rich representation of the original dataset.

The role of vector databases [87] in this context is crucial and deserves emphasis. Traditional relational databases are not optimised for handling the high-dimensional data typical of deep learning outputs. Vector databases, on the other hand, are specifically designed to store, index, and retrieve high-dimensional vectors efficiently. This makes them uniquely suited for dealing with the kind of complex, feature-rich data produced by deep learning models applied to TB-PET datasets. Their ability to perform similarity searches and clustering at scale adds significant value, allowing researchers to quickly and accurately group patients into meaningful categories, such as responders and non-responders to treatments like radioligand therapy and immunotherapy.

Incorporating vector databases into this process facilitates the handling and analysis of these complex embeddings, enhancing the potential of deep learning techniques to discern subtle patterns and correlations within the data. This synergy between deep learning and vector databases can significantly augment the precision and effectiveness of treatments, leading to more personalised therapeutic strategies.

## Charting the future of total-body PET with AI - a call for collaborative innovation

As one reflects on the advancements in TB-PET and its clinical applications, it becomes evident that AI stands at the forefront of enhancing this field. TB-PET’s increased sensitivity and comprehensive diagnostic capabilities, though invaluable, introduce the challenge of managing and interpreting vast amounts of complex data. Here, AI emerges not just as a tool but as a pivotal catalyst in transforming TB-PET from a diagnostic modality to a comprehensive solution for personalised medicine.

The clinical community’s growing interest in TB-PET is primarily driven by its capability for rapid and low-dose imaging. This advancement, however, brings to the fore the need for sophisticated analytical methods capable of managing the resultant data deluge. In this regard, AI-driven tools for segmentation and detection are becoming increasingly crucial. These tools not only streamline the processing of complex datasets but also enable the nuanced interpretation of diverse biomarkers, thus enhancing the overall utility of TB-PET.

Yet, as we advance in integrating AI into TB-PET, challenges persist, notably in ensuring the broad applicability of algorithms across varied clinical scenarios. The development of foundational models, inspired by their success in general vision tasks, is a promising avenue for overcoming these challenges. Such models, adept at segmenting any specified area within medical imaging datasets, hold the potential to revolutionise TB-PET analysis by automating essential processes and improving diagnostic precision. However, the realisation of these foundational models, like MedSAM in radiology, is contingent on the availability of large-scale, diverse datasets and considerable computational resources. The PET imaging field currently faces a gap in available data volumes, with significant initiatives like AUTOPET [29] and HEKTOR [88] providing only a limited number of images. This situation underscores an urgent need within the PET community for a collective effort in data pooling. The prevailing concerns about data protection hindering the sharing of PET images must be re-examined. Given that high-resolution modalities such as CT have been successfully open-sourced, PET imaging should also venture down this path. It is imperative for the community to not only advocate for, but also actively pursue the open source availability of PET data subject to patient privacy regulations that operate in certain jurisdictions.

The creation of a comprehensive normative database from TB-PET scans further exemplifies the need for extensive data pooling. Given the vast variability in human physiology, constructing such a database requires data from diverse and large population samples, something that single sites cannot achieve alone. A normative database, crucial for distinguishing between normal and pathological states, would benefit immensely from a collaborative approach to data collection and sharing. Emulating the open-source successes of radiology could significantly accelerate advancements in TB-PET analysis, paving the way for more personalised and effective patient care.

Building on the momentum of integrating AI into TB-PET and addressing the challenges of data availability and algorithmic applicability, it is essential to also consider the role of AI in enhancing the safety and efficiency of PET/CT imaging. This is particularly pertinent in scenarios like low-dose longitudinal studies, paediatric imaging, or screening, where optimising the CT component of PET/CT imaging becomes crucial. While TB-PET’s increased sensitivity enables inherently low-dose imaging, the radiation dose primarily stems from the CT component, necessitating careful consideration in repeated imaging scenarios. Advancements in AI offer potential solutions to reduce, or in some cases, eliminate the need for CT scans even though CT itself already has a role in some screening approaches and likely itself to provide complementary diagnostic information. Consequently, this approach requires a balanced perspective. CT scans provide essential anatomical details vital for various TB-PET data mining applications, including organ segmentation, multiplexing, and creating normative databases. These tasks depend heavily on CT as it is challenging to work on PET data due to image variability introduced by the tracers. It is crucial to reduce the dose while preserving the critical diagnostic and analytical value that CT imaging brings to TB-PET.

In advancing TB-PET data-mining, the role of Automated Machine Learning (AutoML) is pivotal. AutoML streamlines the process of applying ML algorithms, making it more accessible and efficient. It automates crucial tasks like model selection, hyperparameter tuning, and validation, which are often barriers to effective data analysis in medical imaging. This automation is particularly beneficial in TB-PET, where the data’s multidimensionality can be overwhelming and nuanced. With AutoML and explainable AI paradigms, researchers and clinicians can more readily analyse and interpret complex datasets. Importantly, the progress and acceleration of AI and ML in TB-PET should take cues from the broader AI community, especially regarding the open-source movement. The rapid advancement in AI fields is partly attributed to the community’s commitment to open-sourcing and collaborative development, avoiding the pitfalls of redundant efforts. A prime example is nnU-Net, an open-source framework that has standardised neural network applications in medical imaging. Before nnU-Net, numerous variations of U-Net architectures proliferated, but its introduction streamlined the development process, demonstrating that open-source collaboration can lead to more efficient and effective solutions.

Building on our previous discussions, it is evident there is a pressing need for a unified community initiative to consolidate resources, software, and data in TB-PET and AI. Currently, these elements are fragmented, impeding the pace of progress. Platforms like enhance.pet (https://enhance.pet) serve as a promising model, offering a centralised web hub for data, software, and educational resources. Similarly, the National PET Imaging Platform (NPIP, https://npip.org.uk/) represents another step in the right direction, aiming to create a cohesive framework for advancing PET imaging through shared resources and collective expertise.

## Conclusion

In conclusion, this manuscript has comprehensively explored the transformative role of AI in elevating the capabilities of TB-PET/CT imaging. As we have elucidated, the integration of AI not only augments the efficiency of TB-PET but also unlocks novel applications in both clinical and research settings. However, the journey towards fully realising the potential of AI in TB-PET is not just a technological challenge but a collaborative endeavour. It calls for the dismantling of data silos, the creation of open-source tools, and the establishment of platforms for knowledge and resource exchange.

## Data Availability

Not applicable.
